# Prolonged Repeated Acupuncture Stimulation Induces Habituation Effects in Pain-Related Brain Areas: An fMRI Study

**DOI:** 10.1371/journal.pone.0097502

**Published:** 2014-05-12

**Authors:** Chuanfu Li, Jun Yang, Kyungmo Park, Hongli Wu, Sheng Hu, Wei Zhang, Junjie Bu, Chunsheng Xu, Bensheng Qiu, Xiaochu Zhang

**Affiliations:** 1 Laboratory of Digital Medical Imaging, Medical Imaging Center, First Affiliated Hospital, Anhui University of Chinese Medicine, Hefei, Anhui, China; 2 Department of Acupuncture and Moxibustion, First Affiliated Hospital of Anhui University of Chinese Medicine, Hefei, Anhui, China; 3 Department of Biomedical Engineering, Kyung Hee University, Yongin, Republic of Korea; 4 College of Medical Information engineering, Anhui University of Chinese Medicine, Hefei, Anhui, China; 5 School of Information Science and Technology, University of Science and Technology of China, Hefei, Anhui, China; 6 CAS Key Laboratory of Brain Function & Disease and School of Life Sciences, University of Science and Technology of China, Hefei, Anhui, China; Institute of Automation, Chinese Academy of Sciences, China

## Abstract

Most previous studies of brain responses to acupuncture were designed to investigate the acupuncture instant effect while the cumulative effect that should be more important in clinical practice has seldom been discussed. In this study, the neural basis of the acupuncture cumulative effect was analyzed. For this experiment, forty healthy volunteers were recruited, in which more than 40 minutes of repeated acupuncture stimulation was implemented at acupoint *Zhusanli* (ST36). Three runs of acupuncture fMRI datasets were acquired, with each run consisting of two blocks of acupuncture stimulation. Besides general linear model (GLM) analysis, the cumulative effects of acupuncture were analyzed with analysis of covariance (ANCOVA) to find the association between the brain response and the cumulative duration of acupuncture stimulation in each stimulation block. The experimental results showed that the brain response in the initial stage was the strongest although the brain response to acupuncture was time-variant. In particular, the brain areas that were activated in the first block and the brain areas that demonstrated cumulative effects in the course of repeated acupuncture stimulation overlapped in the pain-related areas, including the bilateral middle cingulate cortex, the bilateral paracentral lobule, the SII, and the right thalamus. Furthermore, the cumulative effects demonstrated bimodal characteristics, i.e. the brain response was positive at the beginning, and became negative at the end. It was suggested that the cumulative effect of repeated acupuncture stimulation was consistent with the characteristic of habituation effects. This finding may explain the neurophysiologic mechanism underlying acupuncture analgesia.

## Introduction

Acupuncture, an ancient healing technique that originated in China, is used by millions of patients in many countries [1]. Continuous use of acupuncture in East Asia and more recently throughout the world has led to the assumption that acupuncture is a relatively effective and safe procedure. However, with the call for evidence based medicine, acupuncture has been tested at the forges of modern medicine [2]. Understanding the physiologic basis of acupuncture is critical to producing reliable results. Proposing and testing ideas about the underlying mechanisms of acupuncture could eventually lead to a real understanding about how acupuncture does work [3]. However, for the present it remains to be seen whether we are dealing with a specific physiological response of the brain to acupuncture, or with non-specific reactions to an undifferentiated stimulus [1]. In recent 20 years, fMRI studies have been extensively conducted to investigate the neurophysiologic mechanism of acupuncture. Although it is generally agreed that the brain and nervous system play a leading role in processing acupuncture stimuli [4], [5], the specific mechanism underlying the therapeutic effects of acupuncture is still under debate. Some researchers [5]–[10] proposed that the deactivation of the limbic-paralimbic-neocortical system was crucial to producing acupuncture’s therapeutic effects while some others [11] argued that these deactivations did not occur reliably and suggested that brain responses to acupuncture were activation-dominated.

To investigate reasons for the varying results of the previous studies, some influencing factors, including expectation [12], acupuncture sensation [13], [14], methodology [15], [16], pathological status [17], and time-variant characteristics [18], [19], were studied. Among them, acupuncture time-variant characteristics should be one of the most important factors based on the following facts. First, the stimulation duration and paradigm in different studies varied; some studies had only one run of acupuncture stimulation for several minutes [20]–[28] while other studies had several runs of stimulation lasting nearly half an hour or longer [6], [8], [9], [29]–[31]. The varied duration and paradigm might lead to the different findings of these acupuncture fMRI studies. Secondly, it is generally accepted in clinical practice that the acupuncture treatment ought to be longer than a few minutes in length and repeated [4], [18], [19], [32], [33]. The cumulative effects of prolonged, repeated acupuncture stimulations in the brain may differ from the instant effects of acupuncture stimulation, and the time-variant characteristics of acupuncture stimulation, which was engaged in differential temporal neural responses in a wide range of brain networks [34], might be the fundamental reason of cumulative effects.

Because of these reasons, prolonged acupuncture stimulation was suggested [4], [19], [32] in order to investigate the cumulative effects of typical clinical application of acupuncture. However, some of questions still remained. What the acupuncture cumulative effect could be? Is it the brain activations or deactivations of some specific brain areas or some natural neurophysiologic process? For the present it remains to be seen whether we are dealing with a specific physiological response of the brain to acupuncture, or with non-specific reactions to an undifferentiated stimulus [1]. Generally speaking, acupuncture is a kind of stimulus [4]. A wide range of experiments on organisms ranging from amoebas to humans demonstrated a natural response of habituation to a variety of external repeated stimulus [35], including the acupuncture stimulation. In 1966, a landmark paper published by Thompson and Spencer [36] clarified the definition of habituation by presenting nine characteristics of habituation. Recently, a group of researchers who study habituation redefined these characteristics based on the 40 years of research adding a new characteristic termed as long-term habituation to the original list of the nine characteristics [37]. Considering the ten characteristics of habituation, we believe that the mechanism of acupuncture analgesia, the most widely accepted and proven application of acupuncture [38], could be reasonably explained through the characteristics of habituation, especially the characteristic of generalization to other stimuli (characteristic #7) and the characteristic of long-term habituation (characteristic #10). Therefore, we hypothesized that the prolonged repeated acupuncture stimulation could produce the habituation effect in the brain response that might be useful for the clinical application of acupuncture.

To validate this hypothesis, we designed an experiment with prolonged, repeated acupuncture stimulations in which three runs of acupuncture fMRI with identical paradigms were performed. Among them, six blocks of acupuncture stimulations were applied with different acupuncture cumulative durations. With the datasets acquired from the three runs of acupuncture stimulation, the cumulative effect of acupuncture stimulation was investigated.

## Materials and Methods

### 1. Subjects

Forty healthy volunteers, 20 males and 20 females ranging in age from 21 to 32 years old, were recruited for this experiment. This study was approved by the Institutional Review Board of the First Affiliated Hospital of Anhui University of Chinese Medicine, and written informed consent was obtained from each participant prior to the experiment. All volunteers were right-handed college students with no history of mental, psychiatric or neurological disorders, drug abuse, or abnormality in brain. Any volunteer who had acupuncture experience in the past three months was excluded.

### 2. Experiment Procedures

Before the experiment, the subjects were asked not to fall into sleep in the process of experiment, and then they were asked to relax and their ears were plugged with cotton balls to reduce audio stimulations. During the entire scanning process, the subjects were also asked to close their eyes and avoid any psychological activities. At the end of the experiment, the subjects were asked whether or not they had fallen asleep, and then answered questionnaires about the sensations experienced on the stimulated acupoints on the both sides. Sensations including soreness, numbness, fullness, aching, spreading and heaviness were scored in four grades (0 = no sensation felt, 1 = mild, 2 = moderate, and 3 = severe).

**Figure 1:**
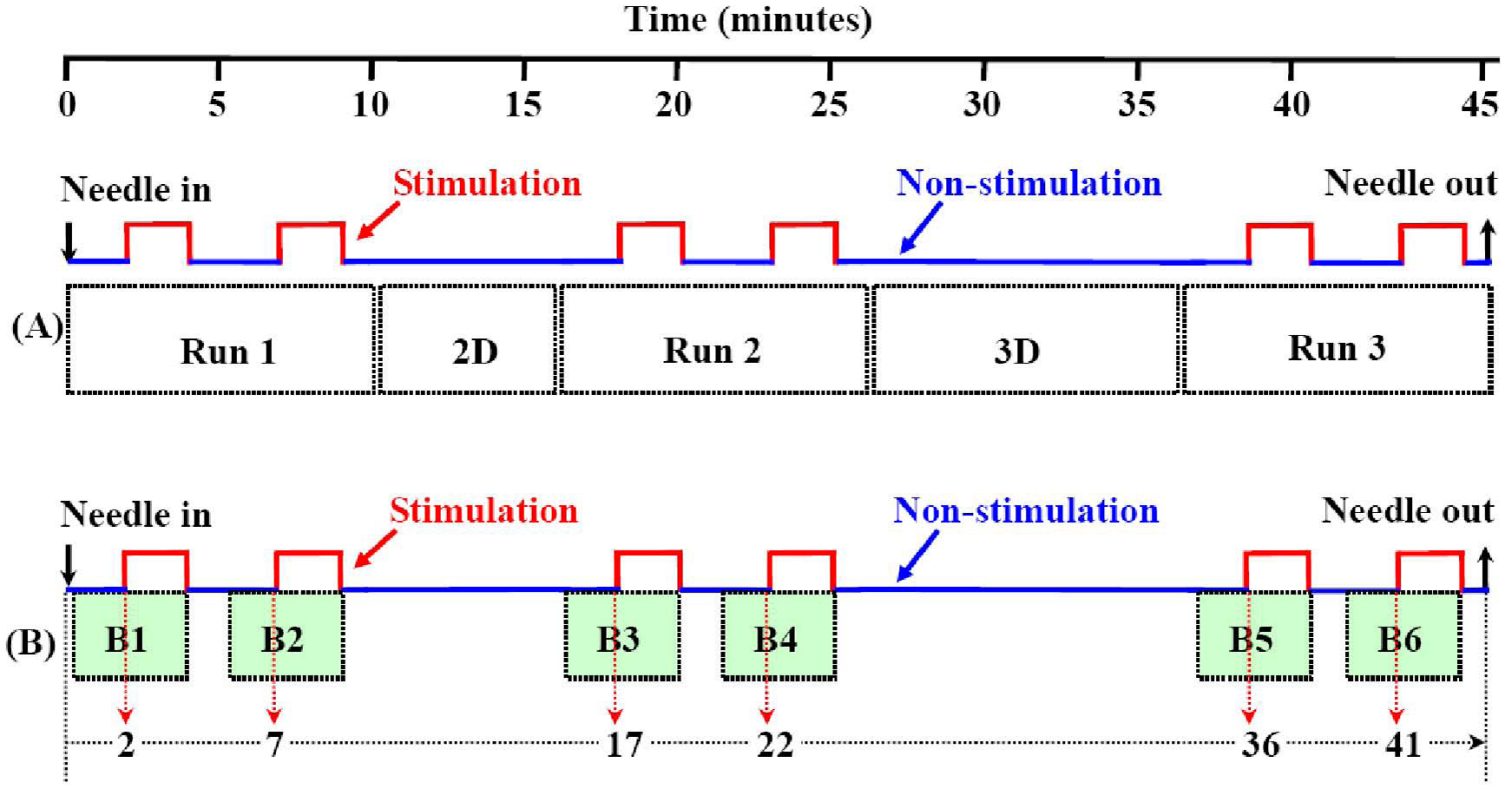
Diagrams of paradigm design and data selection. A: Diagram of paradigm design and data acquisition. From the time of needle-in to the time of needle-out, five series of MRI data were acquired, which were the first run of fMRI (Run 1), 2D anatomical images (2D), the second run of fMRI (Run 2), 3D anatomical images (3D) and the third run of fMRI (Run 3), respectively. Each run of fMRI data acquisition consisted of ten minutes of scan with two blocks of two minutes acupuncture stimulation, and the durations of non-stimulation state were 2 minutes, 3 minutes and 1 minute before, between and after the two blocks of stimulation, respectively. B: Diagram of data selection for each block analysis and definition of covariable of duration. Six blocks of data for block response analysis were B1, B2, B3, B4, B5, and B6, respectively. Each block consisted of two minutes data during stimulation and two minutes data before stimulation (non-stimulation). The covariable of cumulative acupuncture durations, i.e. the time delay from the time of acupuncture needle-in to the beginning of each block of stimulation, were 2, 7, 17, 22, 36 and 41 minutes respectively.

All of the fMRI experiments were completed on a 1.5T whole-body MRI scanner (Symphony; Siemens Medical System, Erlangen, Germany) with a standard head coil. The MR imaging sequences included: (1) T2-weighted MRI to exclude any obvious diseases in the brain; (2) first run of fMRI/EPI-BOLD parallel to the AC-PC line with 36 slices that covered the whole brain with TR/TE/FA 4000 ms/30 ms/90°, FOV 192 mm×192 mm, and matrix 64×64, at 150 time points; (3) T1-weighted 2D anatomical MRI with the same slices of the fMRI with TR/TE 500/12 ms, FOV 230 mm×230 mm, slice thickness/interval 3.0 mm/0.75 mm, and matrix 192×144; (4) the second run of fMRI with the same parameters of the first run of fMRI; (5) T1-weighted 3D anatomical sagittal images with a total of 176 slices that covered the whole brain using a spoiled gradient echo sequence with TR/TE/FA 2100 mm/3.93 mm/13°, FOV 250 mm×250 mm, slice thickness/spacing 1.0 mm/0.5 mm and matrix 256×256, followed by the third run of fMRI with the same parameters of the first run of fMRI (see **Figure 1A**).

### 3. Experimental Paradigm and Data Acquisition

Three runs (six blocks) of acupuncture fMRI were performed, with each block lasting 2 minutes. Following the fMRI, two series of 2D and 3D anatomical MRI, which lasted for about 5 minutes and 9 minutes respectively, were interposed into the three runs of acupuncture fMRI (see **Figure 1A**). The acupuncture stimulation lasted more than 40 minutes from the time of the needle-in to the time of needle-out.

### 4. Acupuncture Manipulation

Before the first run of fMRI, the sterile and disposable acupuncture needles with size of 25 mm×0.30 mm (Suzhou Medical Appliance Factory, China) were inserted into the bilateral acupoints of *Zhusanli* (ST36) at a depth of 15–20 mm. Once the *De-Qi* sensation was elicited, the handles of the needles were connected to an electro-acupuncture machine (Shanghai Huayi Medical Equipment Co., China) at frequency of 2 Hz and intensity of 2 mA. After finishing the third run of fMRI, the needles were disconnected from the electro-acupuncture machine and pulled out from the acupoints. All the operations of acupuncture were performed by a professional acupuncturist.

### 5. Data Analysis

In order to remove the possible influence of head movement, the data with head movement greater than 1 mm or 1° were excluded. As a result, six cases were excluded in this study. The remaining fMRI data involving 34 subjects (18 females and 16 males, age 25.0±2.3 years old, ranging from 22 to 32 years old) was analyzed using the general linear model (GLM). To be comparable with other previous acupuncture fMRI studies, individual data in each of the three runs were first analyzed with GLM, in which the four time points at the beginning of each run were removed. The group analyses of the first run, the second run, the third run and the three runs in total were performed with *t*-test, respectively.

Considering the possible methodological bias with GLM analysis [18], the individual brain responses to each block were analyzed. The data for the block analysis was extracted from the three runs of fMRI data, which were composed of two minutes of stimulation and two minutes of non-stimulation represented by 30 time points for the stimulation state and non-stimulation state, respectively. After removal of 4 time points at the beginning of the non-stimulation, the data for each block analysis was composed of 26 time points of the non-stimulation state and 30 time points of the stimulation state. After that, acupuncture cumulative effects, which referred to the tendency of acupuncture summed effects that could not be demonstrated in a single block of acupuncture stimulation, were analyzed with analysis of covariance (ANCOVA) to find the correlation between the degree of brain responses and the corresponding cumulative duration of acupuncture (hereafter referred as CDA). The covariance of CDA was defined as the duration from the time of needle-in to the beginning of each of the blocks. Here, the CDAs of the six blocks were 2 minutes, 7 minutes, 17 minutes, 22 minutes, 36 minutes and 41 minutes, respectively (see **Figure 1B**).

The activated or deactivated regions in the brain represented the potentiated or attenuated cumulative effects in the course of the six blocks of repeated acupuncture stimulation. In order to find whether the cumulated areas corresponded with the results of GLM analysis in the first block of acupuncture stimulation, the overlapped brain areas were found with intersection between the activated areas in the first block and the cumulated areas in the course of repeated acupuncture stimulation. Using the overlapped areas as the regions of interest (ROIs), the average and standard error of brain response degree of each ROI in the six blocks were calculated. Then, Pearson correlation coefficients were calculated as a measure of the strength and direction of the linear relationship between the average degree of brain responses and the cumulative duration of the acupuncture stimulation.

## Results

**Figure 2:**
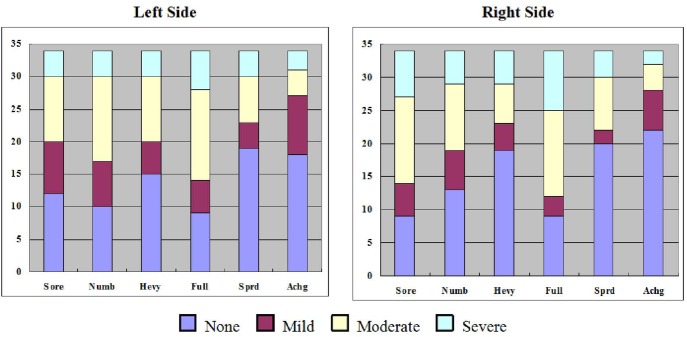
Demonstration of acupuncture sensation composition of different degrees in the both sides of acupoints. The acupuncture sensations were labeled on the x-axis, including soreness (Sore), numbness (Numb), heaviness (Heav), fullness (Full), spreading (Sprd) and aching (Achg). The different degrees of sensations were marked with different colors as shown in the legend. The numbers on the y-axis indicated the cases for each kind of sensation.

**Figure 3:**
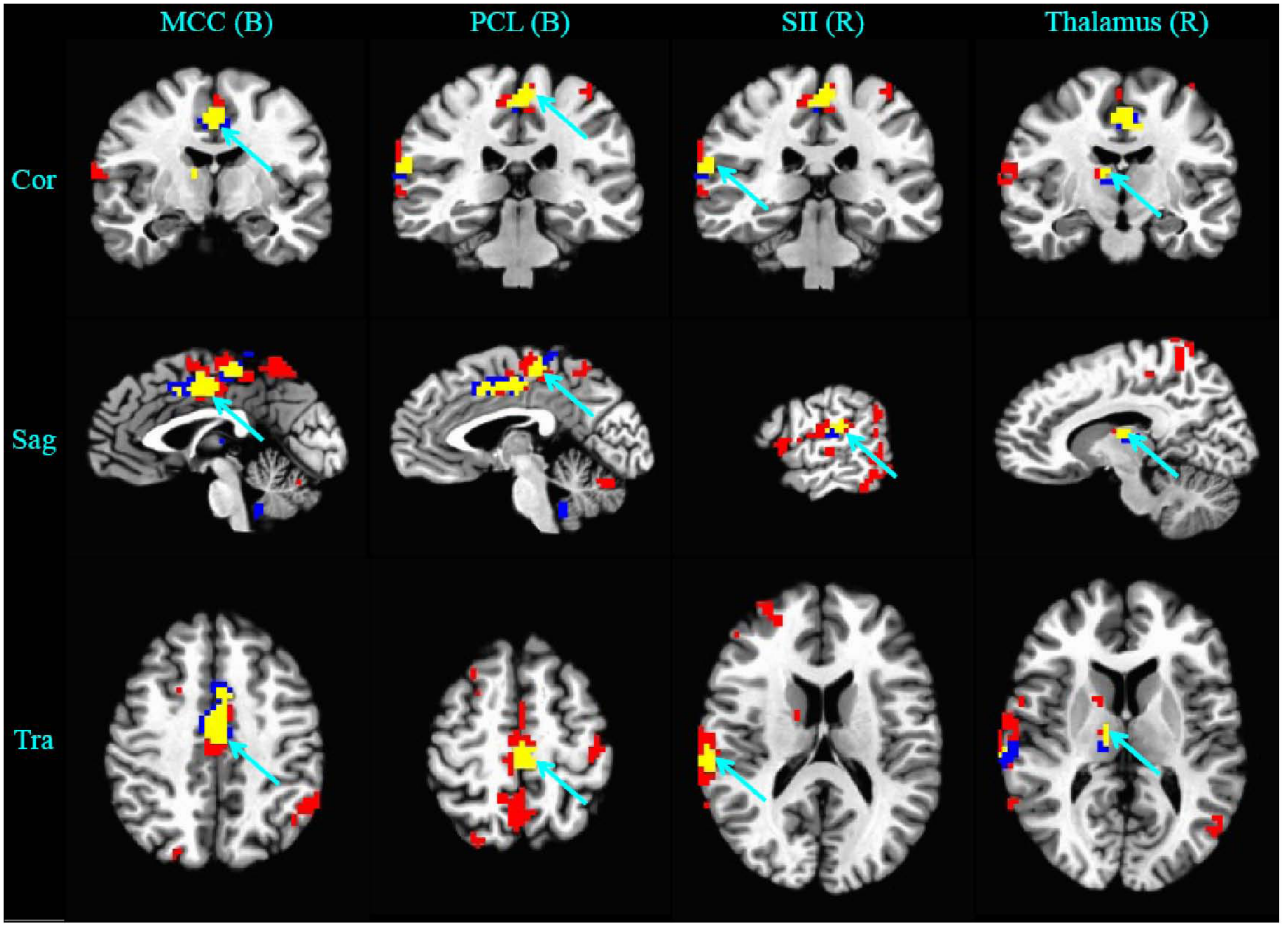
Demonstration of overlapped brain areas (in yellow color) between the activated areas in the first block (in red color) and the habituated areas in the course of six blocks of repeated acupuncture stimulation (in blue color). The threshold was set to *P*< = 0.005, α< = 0.01 (corrected with the Monte Carlo Method). Cor: coronal; Sag: sagittal; Tra: transversal; B: bilateral; R: right; L: left. Overlapped areas included the bilateral middle cingulate cortex (MCC), the bilateral paracentral lobule (PCL), the right SII, and the right thalamus.

Based on self-report, no subjects fell asleep during the experiment. The degrees of *De-qi* sensations such as soreness, numbness, fullness, aching, spreading and heaviness at both sides of acupoints were recorded and presented in **Figure 2**. The brain responses to acupuncture stimulation in the first run, the second run, the third run, and the aggregated runs were quite different (**Figure S1**), and the brain responses to the six blocks of acupuncture stimulation were also extremely varied (**Figure S2**). The brain response in the first block was the strongest. Only positive responses (activations) in the first block were found above the threshold (*P*>0.005, *α* = 0.01, Cluster size = 21, Monte Carlo Method) after multiple comparison correction. These activated areas in the first block included the thalamus, the second somatosensory cortex (SII), the middle cingulate gyrus, the paracentral lobule, the inferior frontal gyrus, the superior frontal gyrus, the precentral gyrus, the precuneus, the inferior parietal lobule, the superior temporal gyrus, the middle temporal gyrus, the fusiform gyrus, and the cerebellum (see **Table 1**, **Figure 3**).

**Table 1:** Activated areas in the first block of acupuncture stimulation.

Regions (BA)	Side	Peak z Value	Coordinate(Talairach)			Volume
			Peak x	Peak y	Peak z	(mm^3^)
Fusiform Gyrus	R	3.81	−55.5	+61.5	−12.5	3753
Middle Cingulate Gyrus(24)	B	4.21	+4.5	+1.5	+41.5	3591
Middle Temporal Gyrus(37)	L	4.43	+52.5	+55.5	−6.5	3483
Superior Frontal Gyrus(9)	R	4.16	−37.5	−40.5	+32.5	3159
Paracentral Lobule (6)	B	4.69	+1.5	+19.5	+56.5	3105
Cerebellar Tonsil	L	4.17	+22.5	+43.5	−48.5	2916
Precuneus(7)	R	3.99	−1.5	+58.5	+17.5	2727
SII(40)	R	4.34	−61.5	+28.5	+17.5	2376
Cerebellum Lobule VIIb	L	4.05	+7.5	+70.5	−21.5	2241
Inferior Parietal Lobule(40)	L	4.42	+43.5	+52.5	+47.5	1782
Superior Temporal Gyrus(42)	R	3.80	−67.5	+16.5	+8.5	1242
Superior Frontal Gyrus (6)	R	3.54	−19.5	−13.5	+47.5	1107
Inferior Frontal Gyrus (45)	L	3.97	+55.5	−16.5	+5.5	1026
Superior Temporal Gyrus(22)	R	4.41	−58.5	−7.5	+2.5	972
Inferior Frontal Gyrus(45)	R	3.93	−52.5	−13.5	+2.5	864
Precentral Gyrus(4)	L	4.59	+37.5	+22.5	+59.5	864
Precuneus(7)	R	3.61	−22.5	+73.5	+53.5	837
Middle Temporal Gyrus(37)	L	4.08	+46.5	+64.5	+8.5	648
Thalamus	R	3.84	−10.5	+13.5	+14.5	621

Note: BA, Brodmann area; L, left; R, right; B: bilater; SII: secondary somatosensory cortex.

With ANCOVA analysis, the brain responses in the course of repeated acupuncture stimulation showed that cumulative effects in five brain areas were all attenuated and no potentiated cumulative effects were found (**Table 2**). Four of the five attenuated areas, including the bilateral middle cingulate gyrus, the bilateral paracentral lobule, the right SII, and the right thalamus, were overlapped with 35% to 71% of the activated areas in the first block of acupuncture stimulation (**Table 3**, **Figure 3**).

**Table 2:** Cumulated areas after repeated acupuncture stimulation.

Regions (BA)	Side	Peak z Value	Coordinate(Talairach)			Volume
			Peak x	Peak y	Peak z	(mm^3^)
Middle Cingulate Gyrus (24)	B	−4.93	−1.5	4.5	41.5	**3888**
Paracentral Lobule (6)	B	−3.76	−1.5	25.5	50.5	**1593**
SII (40)	R	−3.82	−61.5	25.5	17.5	**1053**
Thalamus	R	−3.79	−10.5	16.5	11.5	**729**
Cerebellar Tonsil	L	−3.80	4.5	43.5	−36.5	**702**

Note: BA, Brodmann area; L, left; R, right; B: bilater; SII: secondary somatosensory cortex.

**Table 3:** Overlapped areas between the activated areas in the first block and the cumulated areas and their overlapped ratio.

Overlapped areas	Overlapped volume(mm^3^)	Activated volume(mm^3^)	Habituated volume(mm^3^)	Overlapped ratio
Bilateral Middle Cingulate Gyrus (24)	2403	3591	3888	67%
Bilateral Paracentral Lobule (6)	1134	3105	1593	71%
Right SII (40)	540	2376	1053	51%
Right Thalamus	216	621	729	35%

**Figure 4:**
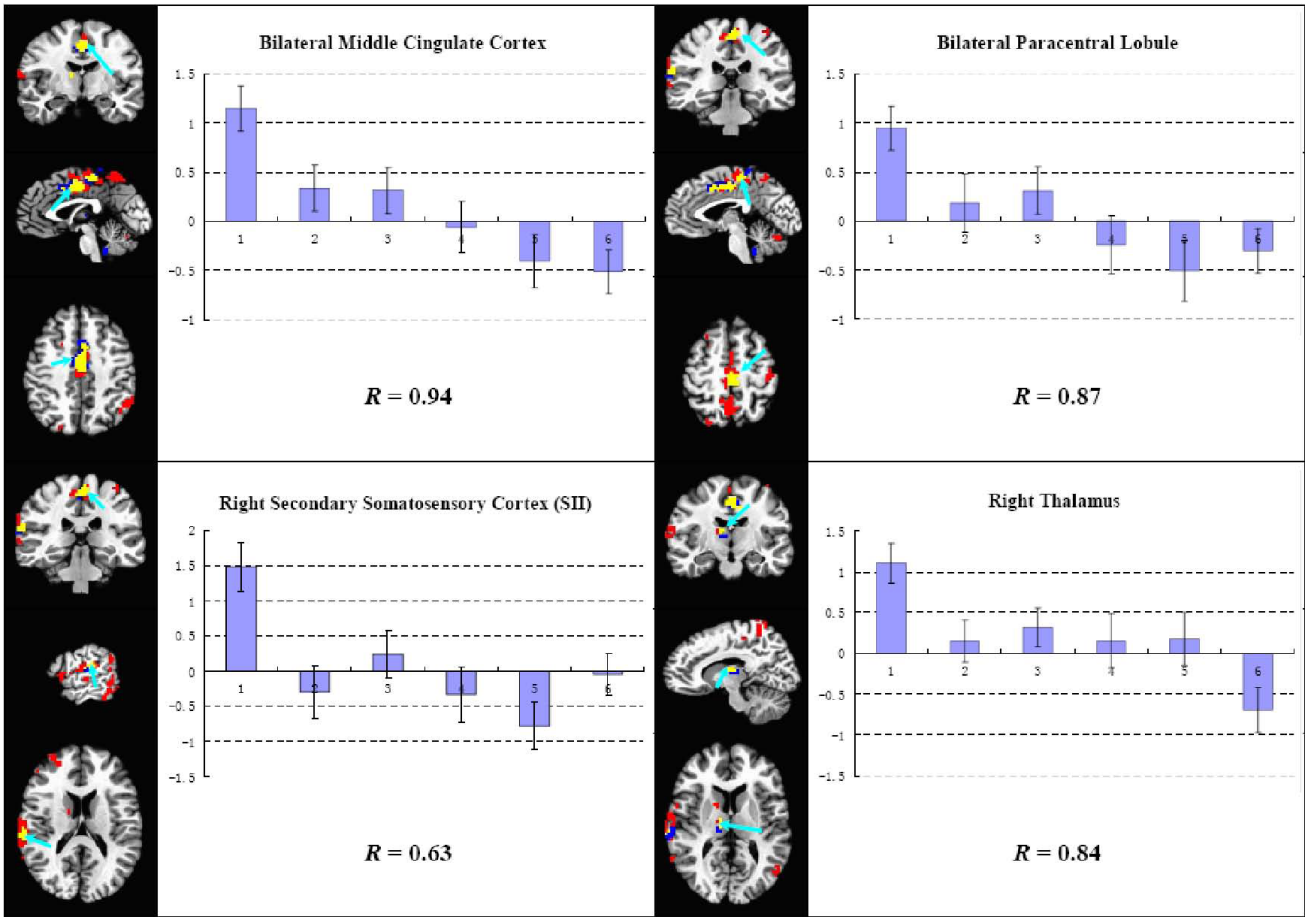
ROIs analysis results of the four overlapped areas between the activated areas in the first block and the habituated areas in the course of repeated acupuncture stimulation. The brain responses to acupuncture stimulation were increasingly decreased as the acupuncture cumulative duration became longer. The characteristic of habitation was bimodal, i.e. positive brain response was found in the first block of acupuncture stimulation, then it began to decrease and brain response became negative in the last (*R*: Pearson’s correlation coefficient).

The brain response in the overlapped areas demonstrated an interesting bimodal characteristic of attenuation, i.e. positive brain response appeared in the first block of stimulation, then the brain response began to decrease and it became negative in the last. The coefficients of correlation between the brain response and the CDA of each block ranged from 0.63 to 0.94 (**Figure 4**).

## Discussion

Our results demonstrated that the brain responses to acupuncture stimulation were time-variant, in which the brain responses to the initial stimulation were the strongest. This finding is consistent with the previous acupuncture fMRI studies [18], [19]. The most interesting finding in this study was that the prolonged repeated acupuncture stimulation induced habituation effects in some pain-related brain areas. In these areas, acupuncture instant effects in the initial stage demonstrated as extensive brain activations and cumulative effects in the process of repeated acupuncture stimulation demonstrated as an interesting characteristic of bimodal habituation, i.e. positive brain response appeared at the beginning of acupuncture stimulation, and then it declined and became negative in the last.

### 1. Acupuncture Cumulative Effects and Habituation Effects

In this study, the brain responses to acupuncture stimulation demonstrated time-variant activations or/and deactivations in different runs and blocks, which was consistent with the time-variant characteristics of brain responses to acupuncture [18], [19]. In order to reduce the influence of the time-variant characteristic on the results of brain responses and avoid the possible methodological problem with GLM analysis [18], we focused on the analysis of brain responses to each block of acupuncture stimulation in this study. The results demonstrated that acupuncture stimulation at the first block resulted in extensive activations in a wide range of brain areas, but no deactivation above the threshold was found. These activations in this areas, including the thalamus, the SII, the middle cingulate gyrus, the paracentral lobule, the inferior frontal gyrus, the superior frontal gyrus, the precentral gyrus, the precuneus, the inferior parietal lobule, the superior temporal gyrus, the middle temporal gyrus, the fusiform gyrus, and the cerebellum, were consistent with most of the previous acupuncture fMRI studies [39]–[64].

The results also demonstrated that all of the cumulative effects were attenuated and no potentiated cumulative effects were found. Interestingly, most of the cumulated brain areas (4 of 5) were overlapped with the activated areas in the first block of acupuncture stimulation, including the middle cingulate cortex, the paracentral lobule, the SII and the thalamus. It suggested that the cumulative effect in these brain areas might reflect some acupuncture related characteristics. In fact, there were some relevant reports about acupuncture that have found consistent results. One report [19] found linearly decreasing time-variant activation in response to both verum and sham acupuncture stimulations in sensorimotor brain regions (SII, posterior insula, premotor cortex) and bimodal time-variant characteristic, i.e. consisting of activation in early blocks, and deactivation by the end of the run, in limbic regions (amygdala, hippocampus, and substantia nigra). Another report [65] showed the similar linearly declined psychophysical response to acupuncture, i.e. the initial acupuncture sensations were the strongest, and then started to drop at 2 minutes and kept decreasing for an hour.

In particular, the results of ROI analysis demonstrated that the brain responses in the overlapped areas were bimodal, i.e. positive response in early stimulation blocks, and negative response in late stimulation blocks. It suggests that the brain response in these areas was a kind of habituation effects. Firstly, this change was consistent with the definition of habituation (characteristic #1), i.e. “given that a particular stimulus elicits a response, repeated applications of the stimulus result in decreased response and the decrease is usually a negative exponential function of the number of stimulus presentations” [35], [37]. Secondly, according to the characteristic #6 of habituation [35], [37], the effects of habituation training may proceed beyond the zero or asymptotic response level, which implies that the brain response could become below-zero or negative. In a word, the habituation effects demonstrated in the process of prolonged repeated acupuncture stimulation should reflect the acupuncture cumulative effects.

### 2. Clinical Implication of the Acupuncture Habituation Effects

Acupuncture cumulative effect was considered as an important factor closely associated with clinical efficiency [4]. Therefore, the key point is to find what the acupuncture cumulative effects reflect. Is it potentiated or attenuated? In this study, with ANCOVA analysis, we did not find any potentiated brain response in the course of repeated acupuncture stimulation, but the attenuated brain response or habituation effect was found in the middle cingulate cortex, the paracentral lobule, the second somatosensory cortex, and the thalamus. Among them, the paracentral lobule located in the upper medial part of the precentral gyrus and the postcentral gyrus (**Figure 3**) was composed of primary motor cortex (MI) and the primary somatosensory cortex (SI) according to the somatotopic map [66]. All of these interested brain areas are significantly related to the process of pain perception. In the human somatosensory system, the contralateral SI is presumed to process and encode the type and intensity of sensory inputs, whereas the bilateral SII responds bilaterally to non-painful and painful somatosensory stimuli, and is believed to perform higher order functions including sensorimotor integration, integration of information from the two body halves, attention, learning, and memory [67]. The MI as a part of motor cortical areas may be related to pain epiphenomena, such as suppression of movement or actual pain-evoked movements themselves [68]. The middle cingulate cortex played an important role in interrupting attention during pain anticipation [69]. Finally, the thalamus is a key relay station for the transmission of nociceptive information to the cerebral cortex, which may hold the key to pain consciousness and the key to understanding spontaneous and evoked pain in chronic pain conditions [70].

All of these cortical or subcortical brain structures demonstrated positive response in the initial stage of acupuncture stimulation. These findings were in accordance with their roles in pain perception because acupuncture stimulation could induce various sorts of pain sensations, such as soreness, aching, or dull pain. Interestingly, the brain responses in these areas began to decrease in the following stimulation and then became negative in the last. As far as the pain perception was concerned, stronger activations of pain-related brain areas might reflect stronger psychophysical ratings of pain [71]. In contrast, the lack of activation in these areas might correlate with weaker pain ratings, and deactivations in these areas were probably able to further reduce the pain rating and increase the pain threshold. In other words, the brain deactivations resulted from the habituation effect in pain related brain areas might play an antinociceptive role. Similar study on heat induced pain was reported by Bingel et al [72]. In their report, pain ratings induced by heat stimulus gradually decreased over time, and the related pain threshold increased over time. The analysis of fMRI data in their report found decreased activity to the thermal stimuli over time (day 1 vs. day 8) and the reduced brain response was found in the pain matrix including thalamus, putamen, insula and SII. This response pattern was consistent with our findings.

On the other hand, it is plausible that even if repeated acupuncture stimulation could result in decrease in pain ratings and increase in pain threshold, it would not necessarily be useful in clinical practice. If acupuncture is to be used as an analgesic, it should meet the following two prerequisites, i.e. the decrease in pain ratings induced by repeated acupuncture stimulation should have the possibility of extending to other sources of pain and the analgesia effects should be sustained for a certain period. In fact, these two prerequisites could be met if the acupuncture cumulative effect could be proved a kind of habituation as previously discussed. It is known that there are ten common characteristics of habituation in total [37]. The most relevant and important ones here are the characteristic #7 and the characteristic #10. The characteristic #7 states “habituation of response to a given stimulus exhibits stimulus generalization to other stimuli”, which implies that the brain habituated to acupuncture stimulation would probably be the neural basis of reducing responses to other similar stimulus, such as acute or chronic pain [73], [74]. Another important characteristic of habituation is characteristic #10, which states, “some stimulus repetition protocols may result in properties of the response decrement that last hours, days or weeks. This persistence of aspects of habituation is termed as long-term habituation”. The characteristic of long-term habituation implies that the analgesia effect, which may result from the generalization characteristic of habituation, has the possibility of lasting for a long time. Therefore, if the acupuncture accumulative effect was of a kind of habituation and the habituation had the general effects, the mechanism of acupuncture analgesia could be easily explained with these common characteristics of the habituation.

### 3. Consistency and Inconsistency with Previous Relevant Studies

Compared with previous fMRI studies on acupuncture, there were some consistency and inconsistency because of the heterogeneity in previous acupuncture fMRI studies. A recent meta-analysis [71] of acupuncture fMRI studies revealed some common activation patterns in the sensorimotor cortical network and deactivation patterns in the limbic-paralimbic-neocortical network following acupuncture needle stimulation. However, another review [11] argued that the reliability of these deactivations was poor. According to the review of Sun et al, brain responses during acupuncture stimulation should be activation-dominated and the deactivations probably resulted from the average of the repeated runs [11]. Our study only found activated areas in the first block of acupuncture after the multiple comparison correction using Monte Carlo method. However, as the stimulation was repeated and the cumulative duration of stimulation increased, the brain responses of those areas, which were positively activated in the first block of acupuncture stimulation, gradually decreased and eventually reversed to deactivation. This finding supported, at least to some extent, the suggestion from Sun et al [11], i.e. the deactivation might result from repeated acupuncture stimulation. However, this standpoint did not imply that acupuncture induced deactivation, which was reported as an important characteristic by many other researchers [5]–[10], was not important. On the contrary, our results indicated that the deactivation was very important, because deactivations induced by prolonged repeated acupuncture stimulation might be the cumulative effect and necessary for acupuncture analgesia as discussed in previous section. Therefore, our results could integrate the previous heterogeneous results in some way, i.e., the deactivation in brain areas following acupuncture stimulation may be time related and it may play an important role in acupuncture cumulative effect but not appear at the initial stimulation stage.

### 4. Limitations in this Study

Although it might be a proper explanation of the mechanism of acupuncture analgesia, the most widely used and most convincingly proved application of acupuncture treatment in clinical practice [4]. However, this presumption could not be convincingly proved with the single result in this study. The first limitation was that we had not adopted sham acupuncture as a control. The main reason was that the objective of this paper was to study the characteristics of brain responses to acupuncture stimulation rather than the specificity of acupoints, i.e. the difference between the true or sham acupoint. Moreover, it was generally agreed that acupuncture, electroacupuncture and transcutaneous electrical acupoint stimulation could be regarded as a continuum of stimulation techniques [4]. As a kind of stimulation techniques, acupuncture stimuli could not be fully distinguished from sensory stimuli, especially when the uncertainty of so called ‘sham acupuncture’ was taken into account [75]. Most importantly, sham acupuncture may be as efficacious as true acupuncture in clinical practice [76]. The second limitation was that the subjects of this study were healthy volunteers rather than patients with chronic pain. Our previous study suggested that brain response to acupuncture of healthy may differ from that of patients [17]. Nevertheless, the ROIs in this study, which were closely related to pain and acupuncture-related brain areas, might provide some support for the presumption. The third limitation in this paper was that acupuncture stimulation was applied in multiple runs in less than an hour rather than in multiple sessions in several days or weeks, because the actual treatment program for acupuncture analgesia usually lasted for at least one or two weeks. Therefore, further longitudinal investigation should be done in the future to provide more evidence for this presumption.

The differences between our finding and another similar finding by Napadow et al [19] included the bimodal habituation which we found not only in limbic regions (middle cingulate cortex) but also in sensorimotor areas (SI, SII and MI) and subcortical areas (thalamus). This inconsistency might result from the difference between the two experimental designs. Our experimental design was a multi-run with six blocks of stimulation, in which each block lasted for two minutes and was irregularly interposed, while their design was a single run design with 31 blocks of stimulation, in which each block lasted for 30 seconds and was regularly interposed. This inconsistency might suggest that the acupuncture related bimodal habituation effect may be dependent of stimulation pattern, which might be a possible reason for heterogeneity of acupuncture effect in clinical practice. In order to conclude that habituation effects might be the mechanism of acupuncture analgesia, further investigations using patients with chronic pain need to be done in the future.

## Conclusion

This study demonstrated that the cumulative effect of prolonged repeated acupuncture stimulation was a kind of habituation effects in pain-related brain areas, where the positive response appeared at the beginning of acupuncture stimulation, and then declined and became negative in the last. It suggested that these increasingly decreased changes of brain response to acupuncture stimulation over time were a kind of habituation effects, a kind of acupuncture cumulative effect. This finding might be useful to explain the neurophysiologic mechanism underlying acupuncture treatment, especially to analgesia, because all brain areas showing acupuncture cumulative effects in current study were related to pain perception. Anyway, further investigations were necessary in order to provide more evidence to support the presumption that acupuncture analgesia was due to the habituation effects of acupuncture stimulation.

## Supporting Information

Figure S1 **Demonstration of activation and deactivation in the first run, the second run, the third run and the three runs in total (p = 0.005, α< = 0.01 corrected with the Monte Carlo Method).** The time-variant characteristic was demonstrated because the brain responses in each run and the total run were quite different.
(TIF)

Figure S2 **Demonstration of activation and deactivation in each of the six blocks (p = 0.05, cluster size = 20, uncorrected).** The time-variant characteristic was showed in the results of block analysis since the brain responses in each block were quite different. The results were not corrected with any method of multiple comparison correction because the results of some blocks failed to pass the Monte Carlo method.
(TIF)
