# Supplementary figures and images for: Prolonged Repeated Acupuncture Stimulation Induces Habituation Effects in Pain-Related Brain Areas: An fMRI Study
Source: PLoS One. 2014 May 12;9(5):e97502. doi: 10.1371/journal.pone.0097502 (PMC4018444; doi:10.1371/journal.pone.0097502)

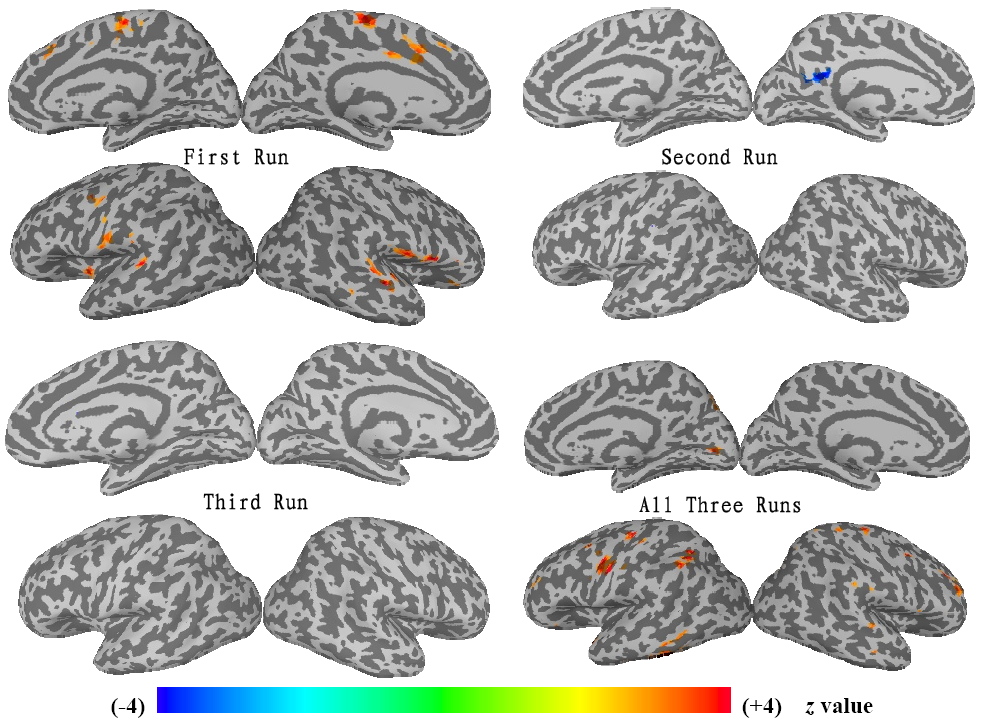

Supplement: Figure S1 — Demonstration of activation and deactivation in the first run, the second run, the third run and the three runs in total (p = 0.005, α< = 0.01 corrected with the Monte Carlo Method). The time-variant characteristic was demonstrated because the brain responses in each run and the total run were quite different. (TIF) [file pone.0097502.s001.tif]

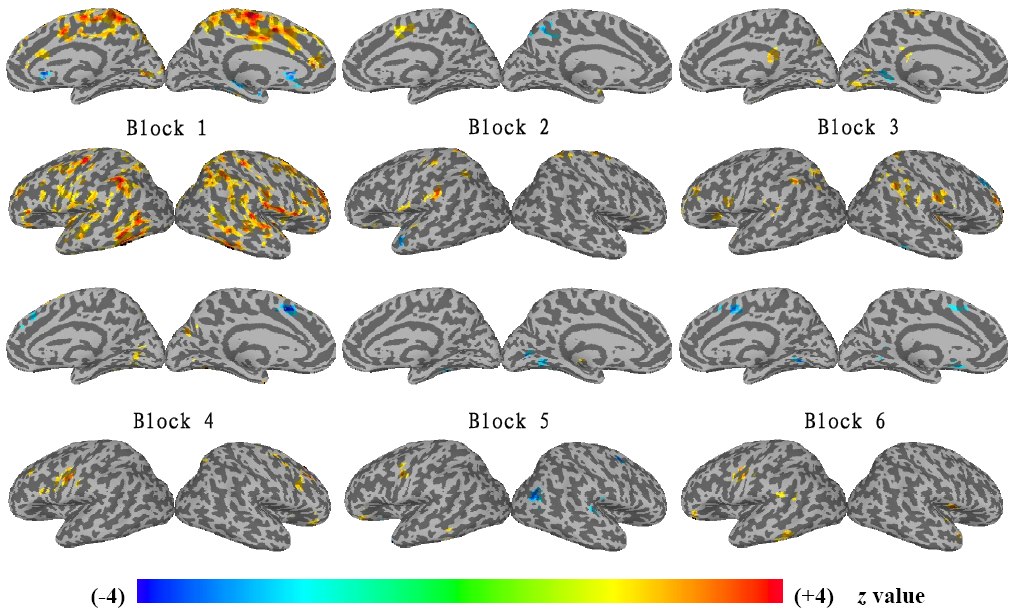

Supplement: Figure S2 — Demonstration of activation and deactivation in each of the six blocks (p = 0.05, cluster size = 20, uncorrected). The time-variant characteristic was showed in the results of block analysis since the brain responses in each block were quite different. The results were not corrected with any method of multiple comparison correction because the results of some blocks failed to pass the Monte Carlo method. (TIF) [file pone.0097502.s002.tif]
